# Lesion level and severity acutely influence metabolomic profiles in spinal cord injury

**DOI:** 10.1093/jnen/nlaf082

**Published:** 2025-07-26

**Authors:** Abi G Yates, Steven Dierksmeier, Yvonne Couch, Timothy D W Claridge, Fay Probert, Daniel C Anthony, Marc J Ruitenberg

**Affiliations:** Department of Pharmacology, Medical Sciences Division, University of Oxford, Oxford, United Kingdom; School of Biomedical Sciences, Faculty of Health, Medicine and Behavioural Sciences, The University of Queensland, Brisbane, QLD, Australia; Department of Pharmacology, Medical Sciences Division, University of Oxford, Oxford, United Kingdom; Acute Stroke Programme, RDM-Investigative Medicine, University of Oxford, Oxford, United Kingdom; Department of Chemistry, Mathematical, Physical and Life Sciences Division, University of Oxford, Oxford, United Kingdom; Department of Pharmacology, Medical Sciences Division, University of Oxford, Oxford, United Kingdom; Department of Chemistry, Mathematical, Physical and Life Sciences Division, University of Oxford, Oxford, United Kingdom; Department of Pharmacology, Medical Sciences Division, University of Oxford, Oxford, United Kingdom; School of Biomedical Sciences, Faculty of Health, Medicine and Behavioural Sciences, The University of Queensland, Brisbane, QLD, Australia

**Keywords:** inflammation, metabolomics, spinal cord injury

## Abstract

Changes in the peripheral metabolome, particularly in the blood, may provide biomarkers for assessing lesion severity and predicting outcomes after spinal cord injury (SCI). Using principal component analysis (PCA) and Orthogonal Partial Least Squares Discriminatory Analysis (OPLS-DA), we sought to discover how SCI severity and location acutely affect the nuclear magnetic resonance-acquired metabolome of the blood, spinal cord, and liver at 6 h post-SCI in mice. Unsupervised PCA of the spinal cord metabolome separated mild (30 kdyne) and severe (70 kdyne) contusion injury groups but did not distinguish between lesion level. However, OPLS-DA could discriminate thoracic level T2 from T9 lesions in both blood plasma (accuracy 86 ± 6%) and liver (accuracy 89 ± 5%) samples. These differences were dependent on alterations in energy metabolites (lactate and glucose), lipoproteins, and lipids. Lactate was the most discriminatory between mild and severe injury at T2, whereas overlapping valine/proline resonances were most discriminatory between injury severities at T9. Plasma lactate correlated with blood-spinal cord barrier breakdown and plasma glucose with microglial density. We propose that peripheral biofluid metabolites can serve as biomarkers of SCI severity and associated pathology at the lesion site; their predictive value is most accurate when the injury level is also considered.

## INTRODUCTION

Metabolomics is a relatively new omics approach, defined as the study of metabolite profiles in cells, tissues, and biofluids. Metabolomics studies combine the use of analytical techniques, such as mass spectrometry or nuclear magnetic resonance (NMR), with multivariate analysis (principal component analysis [PCA] and/or ‘orthogonal partial least squares discriminant analysis’ [OPLS-DA]), to simultaneously measure the concentrations of a constellation of aqueous and/or lipid soluble metabolites. This has allowed for rapid evaluation of a range of metabolic perturbations associated with distinct pathologies, thereby facilitating a more comprehensive understanding of pathophysiology.

Previous studies have used metabolomics to demonstrate that spinal cord injury (SCI) induces a particular metabolite profile in biofluids. Specifically, changes in metabolite profiles have been reported in cerebrospinal fluid (CSF) and plasma/serum of both human patients^1–3^ and in rodent models of SCI,^4–9^ with downstream pathway analysis revealing disturbances in amino acid and lipid metabolism. Interestingly, several of these studies also identified severity-dependent perturbations in at least some peripheral metabolites, indicating their potential as diagnostic and/or prognostic biomarkers.^2^^,^^8^ Consistent with this premise, serum metabolites have been correlated with functional outcomes in a cohort of male SCI patients.^10^

While severity-dependent changes have been reported,^2^^,^^7^ the very early impacts of SCI (<24 h) and also the influence of injury location (ie, lesion level) on the peripheral metabolome remain largely unknown. The putative impact and/or influence of these variables on post-SCI outcomes is increasingly recognized as critical time windows may exist during which early systemic changes may predispose to secondary complications later on.^11^ A dysfunctional and/or differentially activated (ie, by lesion level) sympathetic-neuroendocrine adrenal reflex appears to be a shared contributing factor to several SCI sequalae, including increased infection susceptibility, muscle wasting, and neurogenic heterotopic ossification.^11–13^ As the metabolic impact(s) of adrenal hormones such as corticosterone, catecholamines, and/or (nor)adrenaline are well established,^13^^,^^14^ it stands to reason that SCI-induced changes in these will have associated downstream consequences on the metabolite profiles of peripheral tissues; the degree of these changes may vary based on the extent and/or anatomical level of the lesion.

In this study, we used a clinically relevant mouse model of SCI to investigate the early acute impact of lesion level and severity on the central and peripheral metabolome. We utilized NMR to perform our metabolomics experiments because of its quantitative and nondestructive nature, high accuracy, and reproducibility. Then, we also interrogated whether and how acute changes in peripheral metabolites correlated with pathology and inflammation at the lesion site.

## METHODS

### Animals

Female C57BL/6 mice, 8-12 weeks of age, were obtained from Ozgene ARC. While SCI disproportionally affects men,^15^ female mice were used here for ethical reasons as these animals have a decreased complication risk, lower adverse event rates, and increased post-SCI survival compared to male mice. All animals were housed in a specific pathogen-free facility, under standard diurnal lightening conditions (12 h) with ad libitum access to food and water. Animals were allowed to acclimatize for 4 weeks prior to experiments. All procedures were approved by The University of Queensland’s Animal Ethics Committee (Anatomical Biosciences) and conducted in accordance with the Australian Code for the Care of the Use of Animals for Scientific purposes.

### SCI model

Mice were anesthetized by intraperitoneal injection of tiletamine/zolazepam (50 mg/kg, Virbac) and xylazine (10 mg/kg, Troy Laboratories). Spinal cord injury was simulated as previously described.^16^ Briefly, the skin over the lower back was incised and the paravertebral muscles separated. Anatomical landmarks were used to identify the appropriate target vertebrae. For injury at T9, a dorsal laminectomy was performed to expose the spinal cord, whereas at T2 typically only the spinal disc between T2 and T3 needed removed. The vertebral column was clamped for stabilization and contusive SCI introduced using the Infinite Horizon Impactor (Precision Systems and Instrumentation). At T9, a single midline impact injury with a force of either 30 or 70 kdyne was employed (*n* = 10/severity), to mimic mild and severe lower thoracic contusive SCI, respectively. At T2, animals received either a single midline impact SCI with a force of 30 kdyne, or a double impact SCI (on each side of the midline) with a force of 70 kdyne (*n* = 12/severity), to ensure sufficient damage to the lateral white matter (which contains the descending autonomic pathways) and replicate a similar level of paralysis compared to T9 SCI (Figure S1A). Muscle and skin were then closed with 6-90 polygalactin dissolvable sutures (Ethicon) and Michel wound clips (Kent Scientific), respectively. Following surgery, all mice received a dose of buprenorphine in Hartmann’s sodium lactate (1 mg/kg, subcutaneous, Sigma Aldrich). Given that anesthesia is known to have effects on the peripheral metabolome,^17^ naïve mice (*n* = 10) received anesthesia but did not undergo any surgery to establish baseline results.

### Sample collection and processing

Animals were sacrificed 6 h pos-tinjury to evaluate the impact of SCI severity and lesion level on the blood and tissue metabolome during the very acute phase of injury. Mice were anesthetized with 4% isoflurane and blood was collected by cardiac puncture into a heparinized tube (BD Microtainer). Blood samples were centrifuged at 1000*g*, then 2000*g* (10 min each) to generate platelet-free plasma. Animals were then transcardially perfused with heparinized saline (0.9% NaCl, 10 IU/mL heparin [Pfizer], 2% NaNO_3_), and fresh liver and spinal cord tissue collected from one half of the cohort for metabolomics analysis. Mice in the other half of the cohort were transcardially perfused with Zamboni’s fixative (2% picric acid, 2% paraformaldehyde, pH 7.2-7.4), after which their vertebral columns and spinal cords were excised and postfixed at 4 °C for 48 h. Next, spinal cords were cryoprotected (overnight incubations in 10% and 30% sucrose solution in PBS), embedded in optimal cutting temperature compound (, ProSciTech), and transversally sectioned at 20-μm thickness using the Leica cryostat.

### Immunostaining and quantification

Immunohistochemical staining was performed with 3,3′-diaminobenzidine (DAB) as the chromogen. Endogenous peroxidase activity in the fixed spinal cords was quenched (1% H_2_O_2_) and nonspecific binding was blocked with 10% goat serum for 1 h at room temperature (RT). Sections were incubated overnight at 4 °C with primary antibody (rabbit α-neutrophil, 1:10 000, made in-house; rabbit α-mouse Iba-1, microglia, 1:2000, Abcam); all antibodies were diluted in 1% goat serum. Next, sections were incubated with secondary antibody (biotinylated goat α-rabbit, 1:200, Vector Labs), or biotinylated horse α-mouse IgG (1:200, Vector Labs) to visualize extravasated IgG. Sections were incubated for 2 h at RT, followed by ABC (1:100, ThermoFisher) for 1 h at RT. For visualization, sections were incubated in DAB until satisfactory contrast was achieved; 1% cresyl violet was used as a counter stain. Sections were then dehydrated through graded alcohols (80%, 90%, 2×100%, 5 min each), cleared with xylene (2×5 min) and mounted with DPX.

To quantify immunopositive cells, sections were imaged using a Nikon light microscope with Basler camera and Manual WSIScanner software. Areas were calculated using ImageJ and cells counted using the Leitz Dialux 20 light microscope. The number of cells per mm^2^ was calculated at the lesion epicenter ±1.8 mm in both rostral and caudal direction. All cell counting was performed blinded. IgG staining was used as a surrogate marker for blood-spinal cord barrier (BSCB) breakdown. Sections were imaged as described above and analyzed using ImageJ. Appropriate thresholds were applied and the area of IgG staining per section was measured across the lesion. The volume of extravascular IgG (percent of spinal cord volume) and staining intensity at the lesion epicenter were calculated.

### RNA extraction and qPCR

RNA was extracted from fresh liver samples with the Qiagen RNeasy Mini kit, following manufacturer’s guidelines. Eluted RNA was converted to cDNA using the Applied Biosystems High Capacity cDNA conversion kit. Real-time qPCR was performed on duplicate samples using SYBR green qPCR master mix (PrimerDesign) with the Roche LightCycler 480. All primers were purchased from PrimerDesign, and relative expression of IL-1β, CXCL10, CXCL1, SAA-2 was determined by the 2^-ΔΔCT^ method, normalized to the housekeeping gene RPL13a.

### Metabolomics

Metabolites were extracted from fresh liver and spinal cord samples, as previously described.^18^ Tissues were homogenized with a pestle and mortar on dry ice and combined with 50% acetonitrile (Sigma Aldrich), at a ratio of 1 mg:80 μL. Samples were then centrifuged at 5070*g* for 5 min at 4 °C, after which the supernatants were collected and lyophilized. On the day of analysis, lyophilized samples were resuspended in 600 μL of NMR buffer (75 mM sodium phosphate buffer prepared in D_2_O, pH 7.4). Samples were centrifuged at 2500*g* for 5 min to remove any particulate matter, following which supernatants were transferred to a 5 mm NMR tube. For blood samples, 75 μL of platelet-free plasma was combined with 475 μL of NMR buffer and the mixture was transferred to a 5 mm NMR tube.

All samples were measured using the 700-MHz Bruker AVII spectrometer, operating at 16.4 T, and equipped with a ^1^H(^13^C/^15^N) TCI cryoprobe, as described previously.^19^ Sample preparation was held at 310 K. Spinal cord spectra were acquired with a 1D 1H Nuclear Overhauser Effect Spectroscopy sequence with presaturation delay. Nuclear magnetic resonance spectra of liver samples were acquired using a spin-echo sequence (Carr-Purcell-Meiboom-Gill), 96 data collections, an acquisition time of 1.5 s, a relaxation delay of 3 s, and a fixed receiver gain. Plasma spectra were acquired using a “water suppression with a transverse relaxation filter that eliminates distortions” (WASTED) sequence.^20^

All spectra were phased, baseline corrected, and chemical shifts referenced to the lactate—CH_3_ doublet resonance at *δ* = 1.33 ppm in Topspin 4.0.7 (Bruker). Spectra were then uploaded to ACD/NMR processor academic edition 12.01 (Advanced Chemistry Development, Inc.), and the water signal was removed. The remaining regions were divided into 0.02 ppm width buckets and the absolute value of integral of each spectral bucket was unit variance scaled. Metabolite assignment was performed with references to literature reviews,^21^^,^^22^ HMDB metabolite database,^23^ and 2D total correlation spectroscopy experiments (Table S1).

### Statistical analysis

For analysis of metabolomics data, the integrals of the scaled spectral buckets were imported into R software (R Foundation for Statistical Computing). Multivariate analysis was carried out using in-house R scripts and the *ropls* package. To identify metabolite differences between injury groups, either PCA or OPLS-DA statistical methods were used. Principal component analysis was used with spinal cord data due to smaller *n* numbers; loading plots were generated to evaluate key metabolites driving any cluster separation. OPLS-DA was used with liver and plasma samples to identify specific differences. Injury groups with unequal *n* numbers were corrected, and a 10-fold cross-validation with 100 repetitions was performed resulting in 1000 models. This involved separating the data into a training set (90%) and a test set (10%); the training set was used to build a model and the predictive accuracy of the model was determined on the test set. The mean accuracy, sensitivity, and specificity of the model was compared to that of a separate model created by permuting the group assignments (random chance). Discriminatory variables driving group separation were identified by a high average variable importance (VIP) score. These variables were investigated further by univariate analysis. Correlations between hepatic metabolite levels and expression of acute phase response (APR) genes, as well as correlations between plasma metabolites and spinal cord pathology, were computed with the *cor* function in R; the strength of these relationships was evaluated by Pearson’s correlation coefficients. Receiver operator curve (ROC), area under the curve (AUC), and optimal thresholds were calculated using the pROC package to evaluate the diagnostic potential of SCI-induced metabolite changes.

All other analyses were completed with GraphPad Prism 7 software. The D’Agnostino-Pearson omnibus normality test was applied to all data, and suitable parametric or non-parametric statistical analysis applied subsequently. Unpaired *t*-test, Mann-Whitney test, 1- and 2-way ANOVA and Kruskal-Wallis test were employed. Exploratory post hoc analyses were also performed for instances where no significant interactions between main effect variables were observed, and these results should be hence interpreted with a level of caution. Results were considered significant at *P* < .05 with 95% CI. All quantitative data are expressed as mean ± SEM.

### Transparency, rigor, and reproducibility summary

A sample size of n = 10-12 mice/group was planned and used based on an expected effect size of 0.4 calculated to yield 80% power to detect a significant difference using a 2-tailed Student *t*-test or 2-way ANOVA with a *P*<.05.^3^ Analysis of spinal cord pathology was performed by investigators blinded to the relevant groups.^5^ Fluid biomarker measurements were performed by investigators who were aware of relevant groups. Blood samples were collected by terminal cardiac puncture, between 3 and 6 PM, into a heparinized 21G needle with syringe, and then transferred into a blood tube containing heparin.^6^ Samples were processed by centrifugation to generate platelet-free plasma, which was flash-frozen in liquid nitrogen and stored at −80 °C. On the day of analysis, samples were thawed (0 freeze-thaw cycles) and analyzed at the same time in a single batch. The analyses were validated for research use only. Samples were diluted in NMR buffer (75 mM sodium phosphate buffer prepared in D_2_O, pH 7.4) and mixed by pipetting immediately prior to analysis. All equipment and analytical reagents used to perform fluid biomarker measurements are widely available from commercial company (Sigma, ThermoFisher) and laboratories. Access to the 700-MHz Bruker AVII spectrometer, operating at 16.4 T and equipped with a ^1^H(^13^C/^15^N) cryoprobe, may be available upon request from the Chemistry Research Laboratory at the Department of Chemistry, University of Oxford.^7^ No unexpected events occurred during the study. Correction for multiple comparisons was performed using Sidak’s post hoc test, when appropriate.^10^ Analytic code used to conduct the analyses presented in this study are not available in a public repository; they may be available by emailing the corresponding author for the experimental metabolomics work (F.P.).^13^ All biofluid samples were collected by the investigators and were expended during analysis.^14^

## RESULTS

### Central inflammatory and metabolomic changes following SCI are severity-dependent

**Figure 1.**
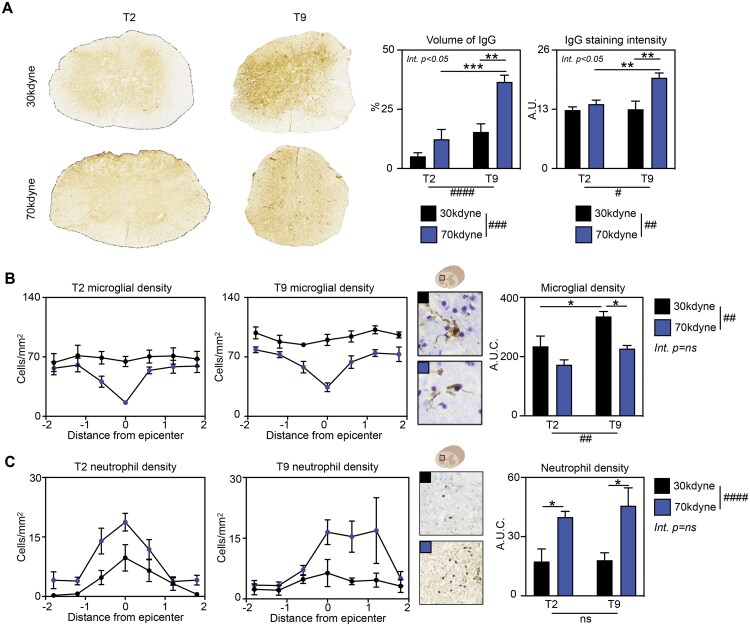
Magnitude of early inflammation and spinal cord pathology is driven by SCI severity. Female mice received either a mild (30 kdyne) or severe (70 kdyne) SCI at the level of thoracic vertebra 2 (T2; high-level lesion), or T9 (low-level lesion). Spinal cord samples were collected 6 h later and the extent of pathology was assessed by immunohistochemistry (*n* = 5-6/group). (A) Volume of IgG as a percentage of total spinal cord volume and average epicenter staining intensity were used as surrogate markers of blood-spinal cord-barrier breakdown. (B) Iba-1 staining was used to determine microglial density. (C) In-house antineutrophil antibody was used to visualize and quantify their infiltration into the injured spinal cord. Data are presented as mean ± SEM, analyzed by 2-way ANOVA. ^#^*P* < .05, ^##^*P* < .01, ^####^*P* < .0001 indicating significant main effect. *P < .05, **P < .01, ***P < .001 indicating significant Sidak’s post hoc test. Abbreviations: Int., interaction; ns, not significant; SCI, spinal cord injury.

First, we evaluated the effect of SCI lesion level and severity on BSCB integrity and acute central inflammation. Spinal cord injury induced a breakdown of the BSCB in a severity-dependent manner (Figure 1A), as evidenced from both the increased volume of IgG staining (2-way ANOVA, severity *P* < .001, level *P* < .0001, interaction *P* < .05) and the overall staining intensity (2-way ANOVA, severity *P* < .01, level *P* < .05, interaction *P* < .05).

Microglia and neutrophil cell densities were calculated to evaluate the early inflammatory response; these cells are among the first to respond during the early acute phase of SCI and known to contribute to lesion development.^24–26^ A decrease in Iba-1^+^ microglial cell density at the lesion epicenter was evident following severe, but not mild SCI at both levels (Figure 1B). We also observed a main effect of injury level, with the microglial cell density being slightly greater at T9 than T2 following mild injury only (2-way ANOVA, severity *P* < .01, level *P* < .01, interaction *P* = .330). The microglial response to SCI is dynamic and, at such an acute time point, the observed decrease in Iba-1^+^ cells at the lesion site is consistent with previous reports attributing this to near-immediate microglial cell death.^27^ Both mild and severe SCI induced recruitment of neutrophils to the lesion (Figure 1C). These cells mostly accumulated at and around the lesion epicenter but were still detectable ±2.0 mm away, particularly with more severe SCI. Area under curve analysis showed a significant main effect of lesion severity, but not level, and no interaction (2-way ANOVA, severity *P* < .0001, level *P* = .590, interaction *P* = .665). Exploratory post hoc tests revealed differences in neutrophil density between 30 and 70 kdyne groups at both injury levels (T2 *P* < .05, T9 *P* < .01).

**Figure 2.**
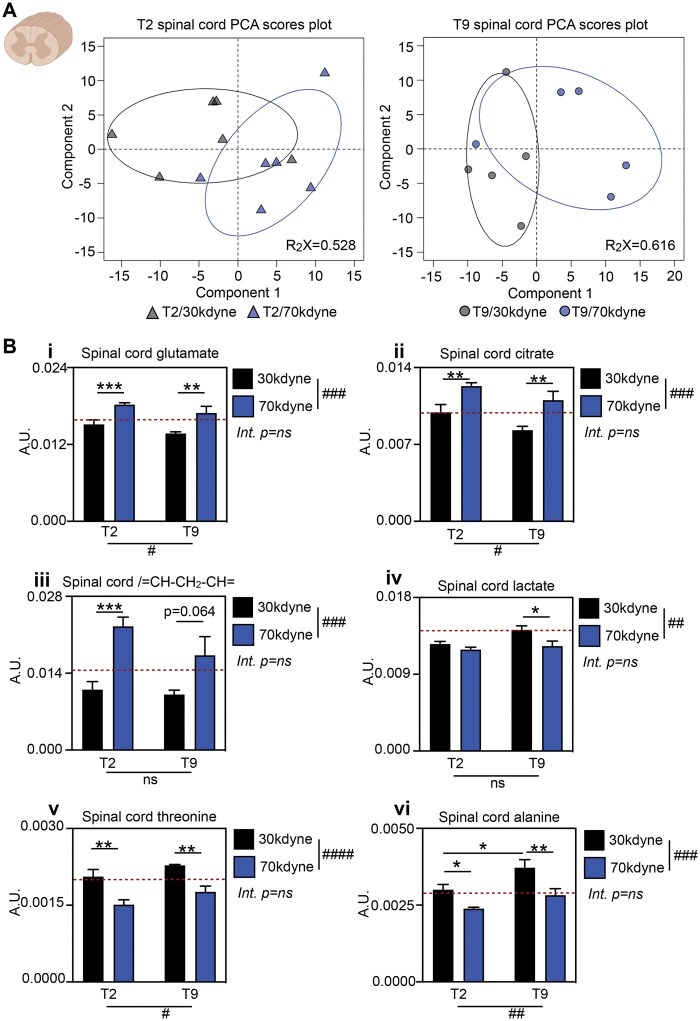
SCI induces metabolic signature within the injured spinal cord that is severity dependent. (A) PCA scores plot of 30 kdyne (black) and 70 kdyne (blue) injuries at the level of T2 (triangles, *n* = 6 group, *R*_2_*X* = 0.528, PC1 = 34%, PC2 = 18%) and T9 (circles, *n* = 5/group, *R*_2_*X* = 0.616, PC1 = 34%, PC2 = 28%). (B) NMR spectral integrals of a selection of metabolites identified as significant discriminators by the PCA loadings—(i) glutamate, (ii) citrate, (iii)/= CH-CH_2_-CH =, (iv) lactate, (v) threonine, and (vi) alanine. Dotted line represents baseline values from anesthetized naive controls (red, *n* = 5). Data are presented as mean ± SEM, analyzed by 2-way ANOVA. ^#^*P* < .05, ^##^*P* < .01, ^###^*P* < .001, ^####^*P* < .0001 indicating significant main effect. *P < .05, **P < .01, ***P < .001 indicating significant Sidak’s post hoc test. Abbreviations: Int., interaction; NMR, nuclear magnetic resonance; ns, not significant; PCA, principal component analysis; SCI, spinal cord injury.

We then performed metabolomics on fresh spinal cord to determine any influence of injury severity and/or level on the central metabolome. Principal component analysis score plots of metabolite profiles revealed modest cluster separation of mild and severe SCI, in the first component, at both T2 (R_2_X = 0.528, PC1 = 34%, PC2 = 18%) and T9 (R_2_X = 0.616, PC1 = 34%, PC2 = 28%) (Figure 2A). To identify significant metabolite differences, we interrogated PCA loadings in the first component (Figure 2B, Table 1). Spinal cord levels of glutamate (Figure 2Bi; severity *P* < .001, level *P* < .05, interaction *P* = .9278), citrate (Figure 2Bii; severity *P* < .001, level *P* < .05, interaction *P* = .7732), and/= CH-CH_2_-CH = lipid resonances (Figure 2Biii; severity *P* < .001, level *P* = .1420, interaction *P* = .2850) were decreased following mild SCI compared to naïve baseline, most consistently so after T9 SCI, and elevated after severe injury. By contrast, spinal cord metabolite levels were decreased after severe SCI (70 kdyne) for lactate (Figure 2Biv; severity *P* < .01, level *P* = .1325, interaction *P* = .3403), threonine (Figure 2Bv; severity *P* < .0001, level *P* < .05, interaction *P* = .8950), and alanine (Figure 2Bvi; severity *P* < .001, level *P* < .01, interaction *P* = .4501). While main effects of lesion level were observed for spinal cord glutamate, citrate, threonine, and alanine, these differences were more modest compared to the effect of injury severity, and there was no significant interaction between these factors. Exploratory post hoc testing further revealed lesion level-dependent differences in lactate (*P* < .05) and alanine (*P* < .05), but only after mild (30 kdyne) SCI. We otherwise observed no cluster separation based on injury level (Figure S1B). Taken together, these data show that early acute changes in the central immunometabolome are largely driven by SCI severity and not location.

**Table 1.** Spinal cord discriminatory metabolites were analyzed by 2-way ANOVA.

Metabolite	Severity (*P*)	Lesion level (*P*)	Interaction (*P*)	Interpretation
Glutamate	.0001***	.0469*	.9278	Spinal cord glutamate levels are trending down with mild SCI and up with severe SCI; some influence of lesion level, but no interaction
Citrate	.0003***	.0218*	.7732	Spinal cord citrate levels are down in mild T9 SCI, but up independently in severe T2 and T9 SCI; no interaction between SCI severity and lesion level
/=CH-CH_2_-CH=	.0002***	.1420	.2850	Spinal cord lipid levels are down in mild SCI and up following severe injury severity, irrespective of lesion level
Lactate	.0058**	.1325	.3403	Spinal cord lactate levels are reduced in both mild and severe T2 SCI, and also in severe T9 SCI. There was no interaction between severity and level
Threonine	<.0001****	.0408*	.8950	Spinal cord threonine levels are affected by injury severity (down with severe SCI) and, to a lesser extent, injury level, independently
Alanine	<.0006***	.0050**	.4501	Spinal cord alanine levels are affected by injury severity (lower in more severe SCI), and to a lesser extent injury level, independently

*P < .05, **P < .01, ***P < .001, ****P < .0001.

### SCI induces distinct metabolic signatures within the periphery

**Figure 3.**
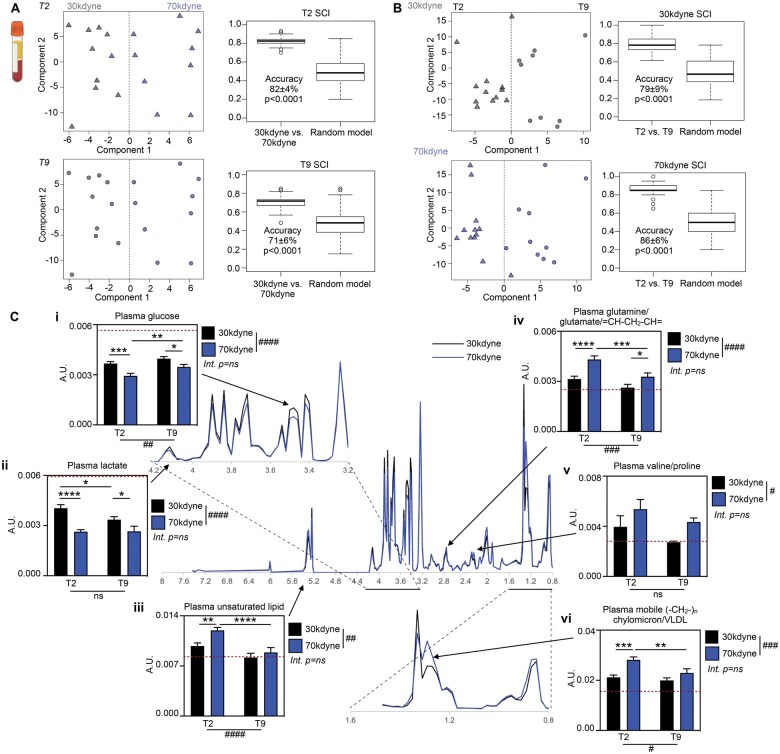
Plasma metabolomes reflect SCI severity and lesion level. (A) OPLS-DA score plots and accuracy of the model discriminating between mild (30 kdyne, black) and severe (70 kdyne, blue) SCI at T2 (triangles, *n* = 12/group) and T9 (circles, *n* = 10/group). (B) OPLS-DA score plots and accuracy of the model discriminating between T2 (triangles) and T9 (circles) after mild (30 kdyne, black) and severe (70 kdyne, blue) SCI. Data are presented as mean ± SEM, analyzed by Student unpaired *t*-test. (C) Average ^1^H WASTED NMR resonance intensity of all plasma samples from animals with mild (30 kdyne, black) and severe (70 kdyne, blue) SCI. Graphs illustrate significant differences in the NMR spectral integrals of a selection of metabolites identified as significant discriminators by the OPLS-DA models—(i) glucose, (ii) lactate, (iii) unsaturated lipid, (iv) overlapping glutamine/glutamate/= CH-CH_2_-CH = resonance, (v) overlapping valine/proline resonance, and (vi) overlapping mobile (-CH_2_-)_*n*_ chylomicron/VLDL resonance. Dotted line represents baseline values from anesthetized naïve controls (red, *n* = 10). Data are presented as mean ± SEM, analyzed by 2-way ANOVA. ^#^*P* < .05, ^##^*P* < .01, ^###^*P* < .001, ^####^*P* < .0001 indicating significant main effect. *P < .05, **P < .01, ***P< .001, ^****^P < .0001 indicating significant Sidak’s post -hoc test. Abbreviations: Int., interaction; NMR, nuclear magnetic resonance; ns, not significant; SCI, spinal cord injury.

Next, we examined the metabolic profile of peripheral tissues. The experiment was sufficiently powered (*n* ≥ 10 per group) to perform supervised multivariate analysis (ie, OPLS-DA) with external cross-validation on specific groups of interest. Metabolomic analysis of plasma confirmed that experimental SCI induced a distinct metabolite signature, the nature of which was dependent on the severity of injury. OPLS-DA was able to discriminate between injury severity at T2 (30 kdyne vs 70 kdyne, Student unpaired *t*-test, accuracy 82 ± 4%, *P* < .0001, *Q*^2^ = 0.627, *R*_2_*X* = 0.692) and T9 (accuracy 71 ± 6%, *P* < .0001, *Q*^2^ = 0.271, *R*_2_*X* = 0.745) (Figure 3A). OPLS-DA was also able to discriminate between lesion level with significant accuracy (T2 vs T9, Student unpaired *t*-test; 30 kdyne accuracy 79 ± 9%, *P* < .0001, *Q*^2^ = 0.459, *R*_2_*X* = 0.641; 70 kdyne accuracy 86 ± 6%, *P* < .0001, *Q*^2^ = 0.574, *R*_2_*X* = 0.621) (Figure 3B), suggesting that the plasma metabolome is also influenced by the location of the injury.

To understand the key metabolites driving these differences between groups, the top VIPs for each model were interrogated by univariate analysis, which revealed a strong SCI severity effect (Figure 3C, Table 2). Specifically, experimental SCI decreased both plasma glucose (Figure 3Ci; severity *P* < .0001, level *P* < .01, interaction *P* = .4067) and lactate (Figure 3Cii; severity *P* < .0001, level *P* = .1276, interaction *P* = .1091) to below naïve baseline levels. This decrease was most pronounced with severe SCI, with plasma levels of glucose and lactate being significantly lower after 70 kdyne SCI compared to 30 kdyne SCI. By contrast, unsaturated lipid (Figure 3Ciii; severity *P* < .01, level *P* < .0001, interaction *P* = .1681), the overlapping resonances (spectral signals from different metabolites that occupy the same frequency) of glutamine/glutamate/= CH-CH_2_-CH = (Figure 3Civ; severity *P* < .0001, level *P* < .001, interaction *P* = .2180), valine/proline (Figure 3Cv; severity *P* < .05, level *P* = .1067, interaction *P* = .8806), and lipoproteins (Figure 3Cvi; severity *P* < .001, level *P* < .05, interaction *P* = .1099) were all elevated after SCI in a severity and/or lesion level-dependent manner. Specifically, and although no significant interactions were observed, exploratory post hoc analysis showed significant lesion-level differences in plasma glucose (*P* < .01), unsaturated lipid (*P* < .0001), glutamine/glutamate/= CH-CH_2_-CH = (*P* < .001), and lipoprotein (*P* < .01) following severe SCI. We also observed a significant difference in plasma lactate (*P* < .05) between T2 and T9, but after mild SCI only. Collectively, these data highlight that plasma metabolites are influenced by SCI severity and level, but that these factors largely act independently.

**Table 2.** Plasma discriminatory metabolites were analyzed by 2-way ANOVA.

Metabolite	Severity (*P*)	Lesion level (*P*)	Interaction (*P*)	Interpretation
Glucose	<.0001****	.0055**	.4067	Plasma glucose levels are negatively affected by injury severity, and to a lesser extent injury level, independently
Lactate	<.0001****	.1276	.1091	Plasma lactate levels are negatively affected by injury severity, but not lesion level
Unsaturated lipid	.0087**	<.0001****	.1681	Plasma unsaturated lipid levels are affected by injury level, trending up in T2 SCI, and to a lesser extent injury severity, independently
Glutamine/glutamate /=CH-CH_2_-CH=	<.0001****	.0006***	.2180	Plasma glutamine/glutamate /=CH-CH_2_-CH= levels are positively affected by both injury severity and injury level, independently of one another, and most profoundly so in T2 SCI
Valine/proline	.0317*	.1067	.8806	Plasma valine/proline levels are trending up with increasing SCI severity; there was no influence of lesion level, and no interaction
Mobile (-CH_2_-)_*n*_ chylomicron/VLDL	.0002***	.0113*	.1099	Plasma lipoprotein levels are positively affected by injury severity, particularly in T2 SCI, but there is no interaction between SCI severity and lesion level

Abbreviation: SCI, spinal cord injury.

*P < .05, **P <.01, ***P <.001, ****P < .0001.

**Figure 4.**
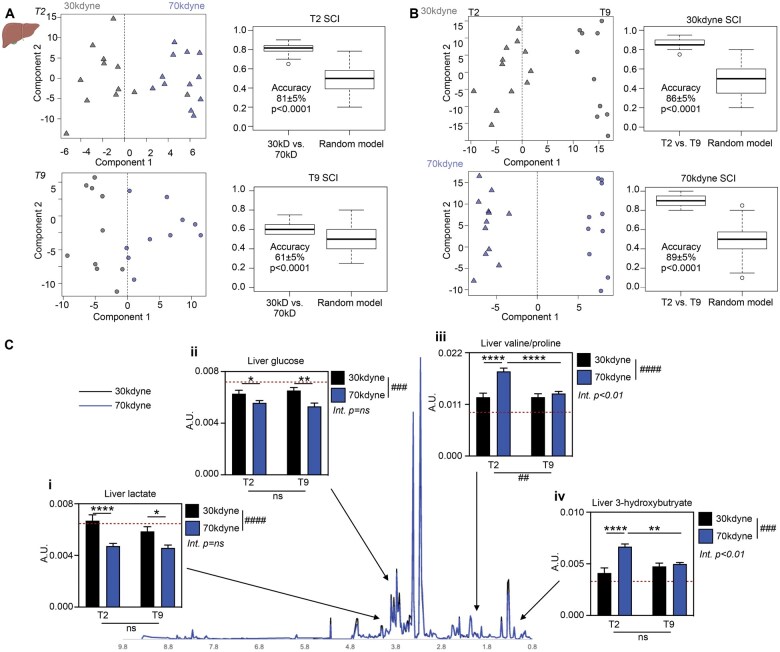
Liver metabolome is dependent on SCI severity and lesion level. (A) OPLS-DA score plots and accuracy of the model discriminating between mild (30 kdyne, black) and severe (70 kdyne, blue) SCI at T2 (triangles, *n* = 12/group) and T9 (circles, *n* = 10/group). (B) OPLS-DA score plots and accuracy of the model discriminating between T2 (triangles) and T9 (circles) after mild (30 kdyne, black) and severe (70 kdyne, blue) injuries. Data are presented as mean ± SEM, analyzed by Student unpaired *t*-test. (C) Graphs illustrating significant differences in the NMR spectral integrals of a selection of metabolites identified as significant discriminators by the OPLS-DA models—(i) lactate, (ii) glucose, (iii) overlapping valine/proline resonance, and (iv) 3-hydroxybutyrate. Dotted line represents baseline values from anesthetized naive controls (red, n = 10). Data are presented as mean ± SEM, analyzed by 2-way ANOVA. ^##^*P* < .01, ^###^*P* < .001, ^####^*P* < .0001 indicating significant main effect. *P < .05, **P < .01, ****P < .0001 indicating significant Sidak’s post hoc test. Abbreviations: Int., interaction; ns, not significant; SCI, spinal cord injury.

The liver plays a crucial role in metabolism and exhibits evidence of metabolic stress after SCI, independent of immobility.^28^ Here, we explored the impact of SCI on the hepatic metabolite profile during the early acute phase of injury. OPLS-DA on liver samples was again able to distinguish between injury severities at both T2 and, to a lesser extent, T9 with accuracies of 81 ± 5% (compared to random model, Student unpaired *t*-test, *P* < .0001, *Q*^2^ = 0.416, *R*_2_*X* = 0.435) and 61 ± 5% (*P* < .0001, *Q*^2^ = 0.159, *R*_2_*X* = 0.611), respectively (Figure 4A). OPLS-DA was also able to differentiate between lesion levels, differentiating mild from severe SCI with 86 ± 5% (*P* < .0001, *Q*^2^ = 0.692, *R*_2_*X* = 0.530) and 89 ± 5% accuracy (*P* < .0001, *Q*^2^ = 0.763, *R*_2_*X* = 0.528), respectively (Figure 4B). Spinal cord injury thus induces specific metabolic signatures in the liver that are dependent on the nature of the lesion. Two-way ANOVA analysis of VIPs driving group separation (Figure 4C, Table 3) revealed main effects of injury severity on liver lactate (Figure 4Ci; severity *P* < .0001, level *P* = .1539, interaction *P* = .3127), glucose (Figure 4Cii; severity *P* < .001, level *P* = .9623, interaction *P* = .2719), valine/proline resonance (Figure 4Ciii; severity *P* < .0001, level *P* < .01, interaction *P* < .01), and 3-hydroxybutyrate (Figure 4Civ; severity *P* < .001, level *P* = .1407, interaction *P* < .01); hepatic lactate and glucose levels decreased after severe SCI relative to mild SCI (and naïve baseline), whereas valine/proline and 3-hydroxybutyrate increased. Significant interactions between injury severity and lesion level for both liver valine/proline and 3-hydroxybutyrate indicate a dependency on these factors with regard to the hepatic metabolome response. Indeed, liver valine/proline and 3-hydroxybutyrate were increased in all SCI groups (compared to naïve baseline), and although they were not influenced by SCI severity at T9, the levels of these metabolites were significantly increased in severe compared to mild SCI at the T2 level (30 kdyne vs 70 kdyne at T2 *P* < .0001 for both metabolites). These data show that SCI induces distinct metabolic signature within peripheral tissues and/or biofluids that are dependent on the lesion level and severity.

**Table 3.** Liver discriminatory metabolites were analyzed by 2-way ANOVA.

Metabolite	Severity (*P*)	Lesion level (*P*)	Interaction (*P*)	Interpretation
Lactate	<.0001****	.1539	.3127	Liver lactate levels are negatively affected by SCI severity, but not lesion level
Glucose	.0002***	.9623	.2719	Liver glucose levels are negatively affected by SCI severity, but not level
Valine/proline	<.0001****	.0024^**^	.0022**	Liver valine/proline levels are positively influenced by injury severity after T2 but not T9 SCI. The significant interaction confirms that the effect of SCI severity is different depending on injury levels, and that the effect of lesion level is dependent on SCI severity
3-hydroxybutryate	.0002***	.1407	.0018**	Liver 3-hydroxybutyrate levels are positively influenced by injury severity after T2 but not T9 SCI. There was no main effect of lesion level, but a significant interaction with SCI severity was still observed

Abbreviation: SCI, spinal cord injury.

**P < .01, ***P < .001, ****P < .0001.

**Figure 5.**
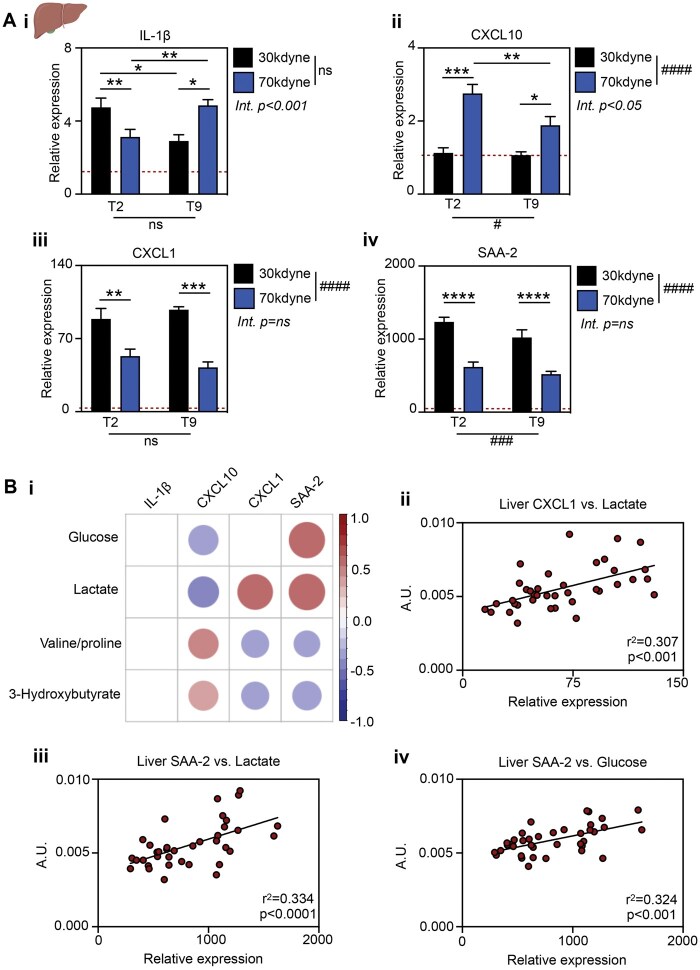
Hepatic inflammatory response to SCI is injury severity and level dependent and correlates with metabolite disruption. Female mice received either a mild (30 kdyne) or severe (70 kdyne) SCI at the level of thoracic vertebra 2 (T2; high-level lesion), or T9 (low-level lesion). (A) Fresh liver was collected 6 h later and expression levels of IL-1β (i), CXCL10 (ii), CXCL1 (iii), and SAA-2 (iv) were determined by qPCR with RLP13a as the housekeeping gene (*n* = 5-12/group). (B) VIP liver metabolites were correlated against hepatic expression of cytokines in the same animal. Significant correlations are shown (i, Pearson’s coefficient, *P* < .05), with the color and size of each dot reflecting the direction of the correlation (positive = red, negative = blue) and value of the coefficient, respectively. Example scatter plots demonstrating significant correlations: (ii) CXCL1 expression vs lactate, (iii) SAA-2 expression vs lactate, (iv) SAA-2 expression vs glucose. Data are presented as mean ± SEM, analyzed by 2-way ANOVA. ^####^*P* < .0001 indicating significant main effect. ^*^P < .05, **P < .01, ***P < .001, ****P < .0001 indicating significant Sidak’s post hoc test. Abbreviations: Int., interaction; ns, not significant; SCI, spinal cord injury.

To explore any relationship between changes in the hepatic metabolome and the APR associated with experimental SCI,^24^ we next performed qPCR on fresh liver samples (Figure 5A). Hepatic expression of IL-1β was increased in all SCI groups above naïve baseline (Figure 5Ai; 2-way ANOVA, severity *P* = .7291, level *P* = .9065, interaction *P* < .001). Following T2 SCI, IL-1β expression was greatest in the mild injury group, and significantly lower with more severe injury (*P* < .01). By contrast, IL-1β expression increased in a severity dependent manner following injury at T9 (*P* < .05). IL-1β expression was significantly higher in mild T2 vs T9 injury (*P* < .05), whereas IL-1β expression was higher after T9 SCI following more severe injury (*P* < .01). These findings demonstrate that injury severity and lesion level have a combined effect on hepatic IL-1β expression, and that both factors must thus be considered during interpretation of results.

Hepatic CXCL10 expression was acutely increased following severe but not mild SCI, and in a lesion level-dependent manner (Figure 5Aii; 2-way ANOVA, severity *P* < .0001, level *P* < .05, interaction *P* < .05). Intriguingly, while SCI caused significant lesion level-independent increases in liver CXCL1 (Figure 5Aiii; injury *P* < .0001, level *P* = .9051, interaction *P* = .2525) and SAA-2 (Figure 5Aiv; injury *P* < .0001, level *P* < .001, interaction *P* = .1187), these changes were most profound in the mild injury groups and were significantly lower following severe SCI. These findings highlight a suppressive effect of SCI severity over the inductions of these APR genes.

We then also compared hepatic expression of cytokines to the observed changes in liver metabolites (Figure 5Bi). IL-1β expression did not correlate with any of the key metabolites impacted by SCI severity and lesion level (refer to Figure 4C). By contrast, hepatic CXCL1 expression exhibited a direct correlation with liver lactate (Figure 5Bi and ii; Pearson’s correlation, *r*^2^=0.307, *P* < 0.001). CXCL1 and SAA-2 were inversely correlated with changes in valine/proline and 3-hydroxybutyrate; SAA-2 further correlated with liver lactate (Figure 5Biii; Pearson’s correlation, *r*^2^=0.334, *P* < .0001) and glucose levels (Figure 5Biv; Pearson’s correlation, *r*^2^=0.324, *P* < .001). Conversely, hepatic CXCL10 expression after SCI positively correlated with changes in valine/proline and 3-hydroxybutyrate resonances and exhibited significant inverse correlations with lactate and glucose (Figure 5Bi and data not shown).

### Plasma metabolome may serve as a useful biomarker for SCI severity

**Figure 6.**
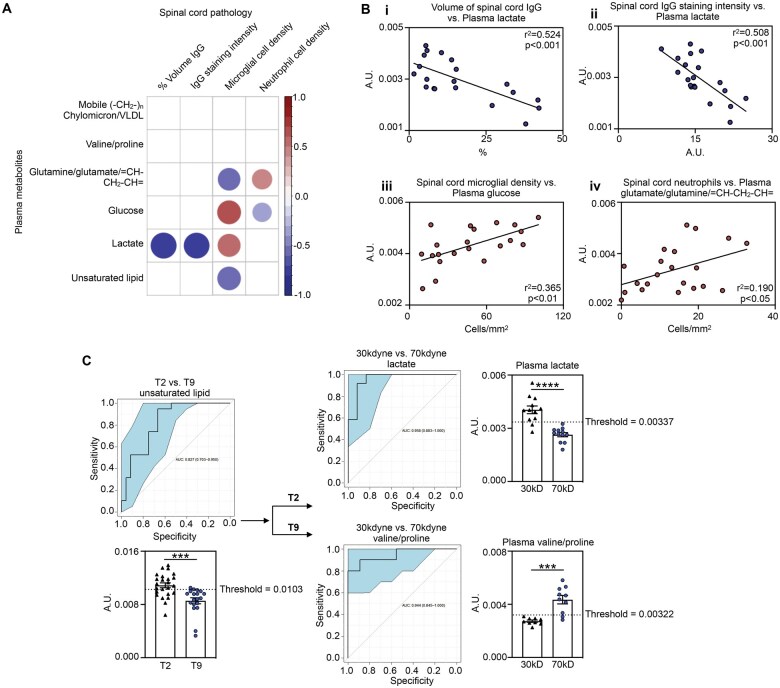
Plasma metabolome correlates with spinal cord pathology and may serve as a biomarker of injury severity. (A) VIP plasma metabolite were correlated against markers of spinal cord pathology in the same animal. Significant correlations are shown (Pearson’s coefficient, *P* < .05), with the color and size of each dot reflecting the direction of the correlation (positive = red, negative = blue) and value of the coefficient, respectively. (B) Scatter plots showing correlations with the greatest significance: (i) plasma lactate vs % volume IgG infiltration and (ii) IgG staining intensity, (iii) plasma glucose vs microglial cell density, and (iv) plasma glutamate/glutamine/= CH-CH_2_-CH = vs spinal cord neutrophils. (C) Proposed decision tree to classify the nature of a spinal cord lesion during the acute phase of injury based on the plasma metabolome. Note that the discriminatory plasma metabolite used to differentiate between mild and severe SCI is dependent on the level of injury. ROC analysis of metabolites determined area under curve (AUC) and the threshold for optimal accuracy. Data are presented as mean ± SEM and analyzed by Student unpaired *t*-test, ***P < .001, ****P < .0001. Abbreviation: ROC, receiver operator curve.

We finally sought to determine how identified key metabolites correlated with established markers of spinal cord pathology in the same animal (Figure 6A). All animals for which both spinal cord pathology and plasma metabolite data were available, independent of injury group, were included in this analysis. Factors with significant correlations mostly had relatively comparable correlation coefficients, although circulating lactate levels were best linked to spinal cord histopathology. Specifically, plasma lactate levels showed strong inverse correlations with both the volume (Pearson’s correlation, *r*^2^=0.524, *P* < .001) and intensity (Pearson’s correlation, *r*^2^=0.508, *P* < .001) of IgG staining in the injured spinal cord (Figure 6Bi and ii), with IgG staining serving here as a proxy for BSCB breakdown. Plasma glucose levels, which were lower in more severe SCI (refer to Figure 3C) directly correlated with microglial density (Pearson’s correlation, *r*^2^=0.365, *P* < .01), and plasma glutamine/glutamate/= CH-CH_2_-CH = with neutrophil presence at the site of SCI (Pearson’s correlation, *r*^2^=0.190, *P* < .05). Thus, plasma levels of lactate, glucose, and glutamine/glutamate/= CH-CH_2_-CH = may serve as proxies for the extent of damage in the spinal cord, as they correlate with established markers of histopathology. We note, however, that the *r*^2^ values for glucose : microglial density and glutamine/glutamate/= CH-CH_2_-CH = : neutrophil density are modest, indicating that any inference of the spinal cord inflammatory response from these plasma metabolites is less reliable.

Having established that information about the nature of the spinal cord lesion can be extrapolated from the plasma metabolome on an individual level, we also explored the extent to which metabolite profiles may be “diagnostic” in terms of both the anatomical level and severity of SCI. Such a single clinical biofluid test could be advantageous for early assessment, subject grouping and/or stratification, and we developed a proof-of-principle decision tree to further explore such an application in future studies. While all metabolites were impressively discriminatory (Table S2), circulating unsaturated lipids were the most discriminatory between T2 and T9 injuries (79% accuracy; Figure 6C). Focusing on injury at T2, plasma lactate differentiated between mild and severe SCI with the greatest accuracy (92%), whereas the overlapping valine/proline resonance was the most discriminatory between severities at T9.

## DISCUSSION

A deep appreciation of metabolite changes following SCI may prove useful to better understand the pathophysiology of this condition, its early management, and/or yield valuable biomarkers for assessing SCI location and severity during the acute stages of injury. Previous studies have already successfully associated metabolite changes in biofluids (plasma, serum, CSF) with pathology in the spinal cord.^2^^,^^7^ However, a putative interaction between lesion level, severity, and the resulting tissue and blood metabolomes very early after SCI (≤6 h) has not been studied in detail. It is well accepted that the anatomical level of injury differentially affects the periphery,^12^^,^^29^^,^^30^ and early critical time windows have emerged during which systemic SCI-induced changes may predispose to secondary complications and/or regenerative failure.^11^ By comparing contusion injuries of different severities and location, we show that SCI acutely induced a robust metabolite signature in the mouse spinal cord, liver, and blood plasma (compared to anesthetized naïve controls), and also that these changes are influenced by lesion level and severity. Causal relationships between altered metabolite levels and SCI outcomes can now begin to be explored.

Neural tissues are known to have high metabolic demand and the spinal cord is no exception. Lactate is an important energy substrate used by the brain and spinal cord to meet metabolic demand and support neuronal function.^31^^,^^32^ During our analysis of the central metabolome, we observed that SCI decreased spinal cord lactate levels in a severity-dependent manner. Under normal homeostatic conditions, astrocytes convert glucose into lactate, which is then shuttled to neurons for metabolic use.^33^ Following injury to the brain or spinal cord, astrocytes quickly become reactive and adopt a proinflammatory phenotype,^34^ a phenomenon that also involves metabolic reprogramming.^35^ Increased glycolytic rates have indeed been reported in astrocytes under inflammatory conditions,^36–38^ and expression of both the M2 isoform of pyruvate kinase^39^ and aldolase C^40^ (key enzymes involved in glycolysis) is increased in reactive astrocytes following SCI. Follow-up studies could investigate if the decrease in intraspinal lactate levels that we observed may have resulted from elevated glial utilization, and whether this contributes to secondary neuronal loss due to a lack of metabolic support.^41^^,^^42^

Analysis of blood plasma samples showed that lactate levels were also lower in the periphery. CNS-peripheral changes in lactate levels appear to be direct consequences of SCI, given that they were correlated with the extent of primary spinal cord damage and associated BSCB breakdown. Glucose levels were also lower in severe compared to mild SCI, and below those of naïve controls. At first glance, these findings appear at odds with the fact that acute stress hyperglycemia is commonly reported in trauma patients, including those with SCI. However, it is worth pointing out that we intentionally used anesthetized healthy control mice for baseline measurements. This allowed us to identify changes occurring in response to experimental SCI, and to separate these from the impact of anesthesia, which in itself is well known to induce various metabolic changes and cause a rise in blood glucose levels.^17^^,^^43^ Regardless, our overall findings indicate that systemic fuel stores appear lower after SCI, and that peripheral lactate administration ought to be investigated further as a potential treatment option for acute SCI. Several studies already show the potential of such an approach, as supplementing lactate during the acute phase of acquired CNS injury can be neuroprotective, improving outcomes from stroke^44^^,^^45^ and traumatic brain injury.^46^^,^^47^ Administration of lactate is thought to be directly effective here by restoring the availability of energy substrates in the CNS.

Consistent with previous reports,^2^^,^^7^^,^^48^ we demonstrate that the peripheral metabolome signature in general is injury severity-dependent. Beyond lactate and glucose, metabolites such as lipids, lipoproteins, and the overlapping valine/proline and glutamine/glutamate/= CH-CH_2_-CH = resonances were all increased in the blood with greater SCI severity. Spinal cord injury also acutely induced a hepatic metabolic signature at 6 h post impact, which is much earlier than previously reported.^30^ The liver plays a critical role in global metabolism and is known to exhibit inflammatory^24^^,^^49^^,^^50^ and lipid disturbances^30^ following SCI. Because of this, we investigated the relationship between metabolite changes and the hepatic APR, which is known to influence spinal cord pathology.^24^^,^^49^ Crosstalk between inflammation and metabolism in the liver is well established, including in the context of SCI. Kupffer cell activation correlates with deficits in lipid clearance, while increased expression of hepatic IL-1β and TNFα coincides with elevated serum alanine transaminase (ALT) levels.^30^ Liver inflammation at the time of injury has further been shown to influence serum fatty acid levels, mesenteric fat, insulin resistance and, most importantly, worsened SCI outcomes.^51^ Metabolic alterations in obesity also exacerbate liver inflammation and functional deficits after SCI.^52^ Our data highlight at least some interactions between hepatic inflammation and metabolic dysfunction in the liver, and future studies could investigate direct linkages between these two phenomena. It is of interest to point out that we did not observe any significant correlations between hepatic IL-1β and SCI-induced changes in liver metabolites, suggesting that expression of some but not all inflammatory mediators of the APR are associated with and/or influenced by the hepatic metabolome.

In terms of lesion location, we found that early peripheral metabolite profiles were influenced by the anatomical level of injury, albeit to lesser extent than injury severity itself. Multivariate analyses of liver and plasma samples were able to discriminate between high- and low-level injuries after both mild and severe SCI. For most metabolites analyzed (eg, plasma glucose and liver 3-hydroxybutyrate), SCI-associated changes were greater after T2 than T9 injury, suggesting that high-level lesions induce a more profound metabolite dysfunction in the periphery. This is certainly consistent with previous observations on hepatic inflammation where T4 SCI in a rat contusion injury model caused more leukocyte recruitment, proinflammatory cytokine expression, and hepatocellular damage than T12.^53^ Loss of brain control over sympathetic innervation/outflow from the spinal cord to peripheral organs in high-level SCI, most notably the spleen and the adrenal glands, is already known to cause severe hormonal and immune-related disturbances.^12^^,^^54^ It is tempting to speculate that these changes,^14^ along with an altered sympathetic drive to splanchnic organs more broadly (ie, including the liver itself) more severely affect systemic metabolism following high-level SCIs,^13^ and future studies could again examine this further. It is nonetheless clear that the anatomical location of the SCI (ie, lesion level) has a significant effect on the peripheral metabolome, the impact of which is dictated further by the severity of injury.

The finding that there are injury level- and severity-dependent effects of SCI on the peripheral blood plasma metabolome offers a number of opportunities. We developed a proof-of-principle decision tree that can now be subjected to further validation to assess the nature of the SCI. We used ROC analysis to evaluate which key metabolites were most discriminatory between different SCI pathologies. For higher level injuries (T2), plasma lactate proved to be the most discriminatory between mild and severe injury, whereas for lower level injuries (T9), the overlapping valine/proline resonance was more accurate. These results confirm that there is an interaction between injury level and severity on the circulating metabolome in our mouse models of SCI. Levels of metabolites in peripheral biofluids thus remain indicative of severity,^2^^,^^8^^,^^48^ but taking into account differentiating metabolites based on the anatomical level of injury may provide a more accurate diagnosis. Future studies should validate and explore the potential of this approach in both animal models of SCI and human patients.

In conclusion, we have shown that SCI induces distinct metabolite changes in the periphery that depend on both the severity and anatomical level of the lesion. The plasma metabolome could therefore serve as a useful and accessible biomarker to assess spinal cord pathology during the acute phase of injury, which could be useful clinically in instances where patients are not able to comply with neurological examination. Future studies should also focus on more longitudinal experiments, to evaluate if early changes in the peripheral metabolome have prognostic value, that is, whether they correlate with and/or can predict the longer term neurological outcome.

## Supplementary Material

nlaf082_Supplementary_Data

## Data Availability

The datasets used and/or analyzed during the current study are available from the corresponding authors on reasonable request.
