# Supplementary material for: Lesion level and severity acutely influence metabolomic profiles in spinal cord injury
Source: J Neuropathol Exp Neurol. 2025 Jul 26;85(1):24–38. doi: 10.1093/jnen/nlaf082 (PMC12744883; doi:10.1093/jnen/nlaf082)
Supplement: nlaf082_Supplementary_Data [file nlaf082_supplementary_data.zip › MS25-076R1 Anthony Supplementary Table 2.docx]

**Supplementary Table 2.** Univariable ROC analysis of plasma metabolites discriminatory between T2 and T9 SCI. AUC = area under the curve.

| **Metabolite** | **AUC** |
| --- | --- |
| Glucose | 0.686 |
| Lactate | 0.601 |
| Unsaturated lipid | 0.827 |
| Glutamine/glutamate/=CH-CH_2_-CH= | 0.726 |
| Valine/proline | 0.601 |
| Mobile (-CH2-)n chylomicron/VLDL | 0.612 |
