# Supplementary material for: Lesion level and severity acutely influence metabolomic profiles in spinal cord injury
Source: J Neuropathol Exp Neurol. 2025 Jul 26;85(1):24–38. doi: 10.1093/jnen/nlaf082 (PMC12744883; doi:10.1093/jnen/nlaf082)
Supplement: nlaf082_Supplementary_Data [file nlaf082_supplementary_data.zip › Supplemental Figure 1.pdf]

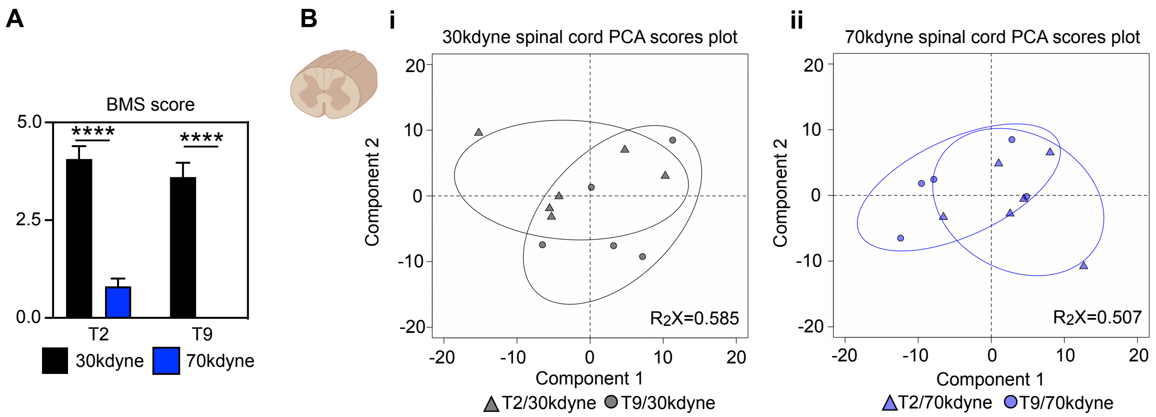

Supplementary figure 1. Spinal cord metabolite profile is not dependent on lesion level. (A) Female mice received either a mild (30kdyne) or severe (70kdyne) SCI at the level of thoracic vertebra 2 (T2; high-level lesion), or T9 (low-level lesion). A double, bilateral impact was used for severe T2 SCI to replicate the extent of paralysis achieved with at T9 injury. (B) PCA score plots for 30kdyne (i, black, $R_2X=0.585$, PC1=35%, PC2=23%) and 70kdyne (ii, blue, $R_2X=0.507$, PC1=34%, PC2=17%) SCI at T2 (triangles, n=6 group) and T9 (circles, n=5/group).
