# Supplementary material for: Lesion level and severity acutely influence metabolomic profiles in spinal cord injury
Source: J Neuropathol Exp Neurol. 2025 Jul 26;85(1):24–38. doi: 10.1093/jnen/nlaf082 (PMC12744883; doi:10.1093/jnen/nlaf082)
Supplement: nlaf082_Supplementary_Data [file nlaf082_supplementary_data.zip › MS25-076R1 Anthony Supplementary Table 1.docx]

**Supplementary Table 1.** Top discriminatory metabolites distinguishing between SCI severity (30kdyne vs. 70kdyne) and lesion level (T2 vs. T9) in the spinal cord, liver, and plasma (from PCA and OPLS-DA analysis).

| **Discriminatory metabolites** | **Chemical shift (ppm) of contributing spectral ‘bins’** |
| --- | --- |
| Glutamate | 2.34…2.36 |
| Citrate | 2.68…2.70 |
| /=CH-CH_2_-CH= | 2.80…2.82 |
| Lactate | 4.10…4.12 |
| Threonine | 3.58…3.60 |
| Alanine | 1.48…1.50 |
| Glucose | 3.50…3.52; 3.68…3.70 |
| Glutamine/glutamate/=CH-CH_2_-CH= | 2.78…2.80 |
| Valine/proline | 2.24…2.26; 2.28…2.30 |
| Mobile (-CH_2_-)_n_ chylomicron/VLDL | 1.28…1.30; 1.30…1.32 |
| 3-hydroxybutyrate | 1.18…1.20 |
| Unsaturated lipid | 5.30…5.32 |
